# De‐Escalation in United Kingdom Inpatient Mental Health Care: A Structured Critical Narrative Synthesis of People, Capability and Context

**DOI:** 10.1111/inm.70364

**Published:** 2026-09-23

**Authors:** Ndukwe Walter Ugwuocha, Simon Neilson, Alexander Challinor, Chengeto Shoko, Deborah Aluh, Oladayo Bifarin

**Affiliations:** ^1^ Mersey Care NHS Foundation Trust Liverpool UK; ^2^ School of Allied Health Professions and Nursing, Institute of Population Health University of Liverpool Liverpool UK; ^3^ Faculty of Health Sciences University of Liverpool Liverpool UK; ^4^ School of Health, Science and Society University of Greater Manchester Bolton UK; ^5^ Department of Clinical Pharmacy and Pharmacy Management University of Nigeria Nsukka Nsukka Nigeria; ^6^ Faculty of Health, Innovation, Technology and Science Liverpool John Moores University Liverpool UK

## Abstract

Aggression and violence remain persistent challenges in inpatient mental health services, with significant implications for patient safety, staff wellbeing, therapeutic relationships and the continued use of restrictive practices. Although de‐escalation is widely promoted as a first‐line, non‐coercive response to distress and behavioural escalation, its implementation remains inconsistent and the evidence base is fragmented across relational, educational, organisational and policy literatures. This paper reports a structured critical narrative synthesis of United Kingdom‐relevant empirical, theoretical, policy and practice literature examining de‐escalation, staff training, restrictive‐practice reduction and organisational responses to aggression and violence in adult inpatient mental health care. Literature published between January 2014 and March 2024 was identified through searches of Scopus, CINAHL, PubMed/MEDLINE, ProQuest and Web of Science, supplemented by reference‐list screening and targeted grey‐literature searches. Sources were selected for their relevance to adult inpatient mental health settings and their contribution to understanding how de‐escalation is enacted, supported and sustained. Given the heterogeneity of the evidence, design‐appropriate appraisal principles informed by the Critical Appraisal Skills Programme and Mixed Methods Appraisal Tool were used to support interpretation. The synthesis identified three interdependent domains shaping de‐escalation practice: People, Capability and Context (PCC). Therapeutic relationships, communication, emotional intelligence and service‐user involvement underpinned early recognition of distress and relationally attuned responses. Workforce capability was strengthened through simulation, reflective learning, team rehearsal and practice‐proximal evaluation. Organisational context, including staffing, leadership, ward environment, safety culture, equity safeguards and wider structural determinants, shaped whether de‐escalation could be enacted consistently and safely. These findings generated the PCC framework as a practice‐facing model that conceptualises de‐escalation as an emergent practice arising from alignment between relational processes, workforce capability and organisational conditions. The framework offers a practical basis for guiding restrictive‐practice reduction, quality improvement, equity‐sensitive monitoring and future empirical research in inpatient mental health care.

## 1 Background

Aggression and violence in inpatient mental health services are persistent and well‐documented threats to both safety and recovery (Goodman et al. 2020; Renwick et al. 2016). Restrictive practices, including physical restraint and seclusion, carry significant ethical, legal and psychological costs and risk eroding therapeutic relationships (Kuivalainen et al. 2017; Wilson et al. 2017). In this context, national policy directives in the United Kingdom (UK) have increasingly prioritised the reduction of restrictive practices, positioning de‐escalation as a central, first‐line intervention (Dixon and Long 2022). De‐escalation is commonly understood as a set of verbal and non‐verbal strategies intended to reduce distress, preserve dignity and prevent behavioural escalation without recourse to restrictive practices (Accinni et al. 2021; Hallett and Dickens 2017; Price et al. 2018). Despite this policy emphasis, de‐escalation practice remains fragmented and inconsistently implemented across inpatient settings. This paper therefore advances a scholarly position that brings together these disparate epistemic strands and argues for a coherent, practice‐grounded framework capable of clarifying what should be standardised at a national level and what must remain responsive to local context, to support reliable, non‐coercive inpatient care.

### 1.1 Implementation Gaps in De‐Escalation Training and Organisational Frameworks

Despite widespread endorsement of de‐escalation training across inpatient mental health services, implementation remains uneven and variable. Foundational work has outlined relational models of de‐escalation and identified key interpersonal and environmental factors shaping conflict and containment on inpatient wards (Bowers et al. 2011; Price and Khubchandani 2016). However, this knowledge has not been translated into a robust national framework in the UK capable of standardising core competencies, team processes and environmental enablers. As a result, de‐escalation training, while now widespread, appears to remain heterogeneous in scope, duration and pedagogy, producing substantial variation in what staff are taught, how training is delivered and whether skills are reliably transferred into everyday practice. This variation reflects a conceptual limitation in understanding de‐escalation, with difficulties seen as individual skill gaps rather than evidence of a complex practice shaped by workforce, relationships, ward culture and organisational context.

### 1.2 Limitations of the Evidence Base and System‐Level Determinants

Although the wider literature on Reducing Restrictive Practices (RRP) is substantial, evidence regarding the sustained, long‐term impact of de‐escalation training on incident frequency and restraint use remains equivocal (Hammer 2015; Long et al. 2016). Existing studies have generated valuable insights into factors associated with aggression, violence and restrictive practices; however, these determinants are frequently examined in isolation. Staffing levels, ward design, organisational culture, leadership and training are commonly treated as discrete variables rather than as interacting influences operating within a complex adaptive system (Bowers et al. 2009; Goodman et al. 2020). As a result, the mechanisms through which these factors combine to support or undermine de‐escalation remain insufficiently understood. This limitation is clear as broader structural factors such as poverty, housing insecurity, social exclusion and systemic disadvantage shape distress, crisis and mental ill‐health but are rarely included in inpatient de‐escalation models (Yearby 2020).

Literature mainly focuses on ward‐level interventions, with little attention to broader social, organisational or policy factors affecting patient acuity and care delivery. However, when attention is narrowed specifically to de‐escalation, the evidence base becomes further fragmented. While some studies report positive outcomes associated with de‐escalation training (Long et al. 2016), evaluations frequently provide limited insight into the active components, underlying mechanisms or contextual conditions required for such interventions to be effective and sustained over time (Barr et al. 2022). Emerging evidence also suggests that behavioural contagion (i.e., whereby distress, aggression or disruptive behaviour may influence the behaviour of others within a shared social environment) and temporal clustering (i.e., whereby episodes of conflict occur at predictable times or under recurring operational conditions) are important features of inpatient mental health settings (Baker et al. 2024; Berry et al. 2016). These patterns limitations indicate escalation is shaped by individual behaviour and by interpersonal, organisational and environmental influences. Consequently, the effectiveness of de‐escalation is highly sensitive to timing, relational dynamics, team responses and contextual cues. Although Daguman et al. (2026) conceptualise de‐escalation as a relational, adaptive and collective nursing practice, this framing remains largely context specific. There is therefore limited empirical evidence demonstrating that de‐escalation is consistently understood or operationalised as an emergent practice arising from the dynamic alignment of relational processes, workforce capability and organisational context.

### 1.3 Core Components of Effective De‐Escalation Practice

Drawing on the conceptual foundation established above, the literature identifies several core components of effective de‐escalation practice. Relational practice, therapeutic communication and staff self‐regulation are repeatedly highlighted as central to successful de‐escalation (Johnston et al. 2022; Lavelle et al. 2016). Evidence also suggests that training must be delivered with consistency and evaluated close to practice to support skill retention and transfer (Brenig et al. 2023; Jalil et al. 2020). In parallel, organisational conditions, including staffing levels, leadership practices and the design and use of physical space, strongly enable or inhibit the timely use of de‐escalation strategies (Goodman et al. 2020; Heffernan et al. 2024).

Within this context, de‐escalation has become established as a core, first‐line intervention aimed at managing aggression and potential violence without recourse to restrictive practices (Renwick et al. 2016). De‐escalation encompasses a range of psychosocial skills intended to reduce anxiety, distress and anger, ideally preventing escalation to containment (Price and Baker 2012). Effective practice involves creating conditions for safe engagement, using clear and respectful communication, clarifying and addressing concerns, conveying empathy and regulating staff emotional responses under pressure (Finch et al. 2022).

### 1.4 International Models and Emerging Evidence

Despite strong face validity and consistent endorsement in national and international guidance, the empirical evidence underpinning de‐escalation practices and training remains uneven. A Cochrane review identified no randomised controlled trials isolating specific de‐escalation approaches for working‐age adults, reflecting the methodological challenges inherent in evaluating complex, relational interventions (Daguman et al. 2026). De‐escalation is typically embedded within multi‐component programmes, making its discrete contribution difficult to quantify (Frohlich et al. 2018).

Nevertheless, international experience provides important indications of promise. German studies identify staff education and regular training as among the most effective measures for reducing restraint and violence, particularly when aligned with organisational change and leadership within models such as Six Core Strategies, Safewards and the Weddinger Model (Knauf et al. 2024; Steinert et al. 2010). Similarly, controlled implementation work in Slovenia reported a 73% reduction in aggressive incidents following structured training in verbal and non‐verbal de‐escalation (Celofiga et al. 2022). While clinically compelling, such studies offer limited knowledge into underlying mechanisms through which de‐escalation operates, contextual conditions required for success or transferability to more complex and resource‐constrained inpatient environments.

Although positive outcomes have been reported across a range of de‐escalation interventions and restrictive‐practice reduction initiatives, the interaction between relational practice, workforce capability and organisational context interacts to influence outcomes remains poorly theorised. Emerging national approaches, such as Norway's Management of Aggression Programme (MAP), further demonstrate the potential of coordinated strategies delivered at scale (Rognmo and Bugge 2025). However, while these models provide valuable guidance on what organisations should implement, they offer less explanation of why interventions succeed in some contexts and not others or how relational, capability and contextual factors combine to support non‐coercive care in routine practice. Although these models have contributed substantially to reducing restrictive practices, they tend to focus on specific interventions, programmes, organisational strategies or training approaches (Brathovde 2021; Goulet et al. 2017). Less attention has been paid to how relational practice, workforce capability and organisational conditions interact to determine whether de‐escalation can be enacted successfully in routine care (Daguman et al. 2026; Tulloch et al. 2022).

### 1.5 Aims

The first aim for this paper is to refine a practice‐grounded account of how de‐escalation and staff capability development can best mitigate aggression and violence in UK inpatient mental health settings. The second aim is to extend this account into practicable guidance for clinical teams, service managers and policymakers, with particular attention to issues of equity and the wider structural determinants that shape experiences of risk and safety. In doing so, the paper seeks to support clinicians, service leaders and policymakers to move beyond fragmented interventions towards a more integrated approach to de‐escalation in inpatient care.

### 1.6 Design

This paper reports a structured critical narrative synthesis designed to generate conceptual clarity and practice‐facing knowledge from a diverse evidence base. The approach combines systematic search, screening, appraisal and synthesis procedures with interpretive analysis, enabling transparent engagement with empirical, theoretical, service evaluation, review, conceptual and policy‐informed literature. This design was selected because de‐escalation in inpatient mental health care is a complex relational, educational and organisational practice that cannot be fully understood through intervention‐effectiveness evidence alone. Rather than reducing the evidence to a single estimate of effect, the synthesis examines how de‐escalation is understood, enacted and sustained across different inpatient contexts.

## 2 Method

A structured search was undertaken to identify literature published between January 2014 and March 2024. This period was selected to capture contemporary evidence produced during increasing policy and clinical emphasis on reducing restrictive practices. Searches were conducted in Scopus, CINAHL, PubMed/MEDLINE, ProQuest and Web of Science, supplemented by targeted searches of grey literature and reference list screening. This approach was chosen to ensure breadth of coverage across heterogeneous evidence types, including qualitative and quantitative studies, service evaluations, policy documents and professional guidance, rather than to achieve exhaustive systematic retrieval (See Appendix A). Where database functionality allowed, searches were limited to English‐language literature, adult populations and publications between January 2014 and March 2024.

### 2.1 Eligibility Criteria

Sources were eligible for inclusion if they addressed de‐escalation, staff training, restrictive‐practice reduction, aggression, violence or organisational responses to conflict in adult inpatient mental health settings. Eligible settings included acute inpatient mental health wards, psychiatric intensive care units, forensic mental health services, secure services and inpatient rehabilitation settings. Studies and papers were included where they contributed empirical, theoretical, policy or practice insight relevant to understanding de‐escalation in inpatient mental health care. Sources were excluded if they focused primarily on community mental health services, supported living, emergency departments, general medical wards, paediatric settings, dementia care, autism‐specific services, learning disability services or aggression arising primarily from neurological or organic conditions. Non‐English publications were excluded. Papers were also excluded where the intervention, setting, population or relevance to inpatient mental health de‐escalation was unclear.

### 2.2 Screening and Selection

**FIGURE 1:**
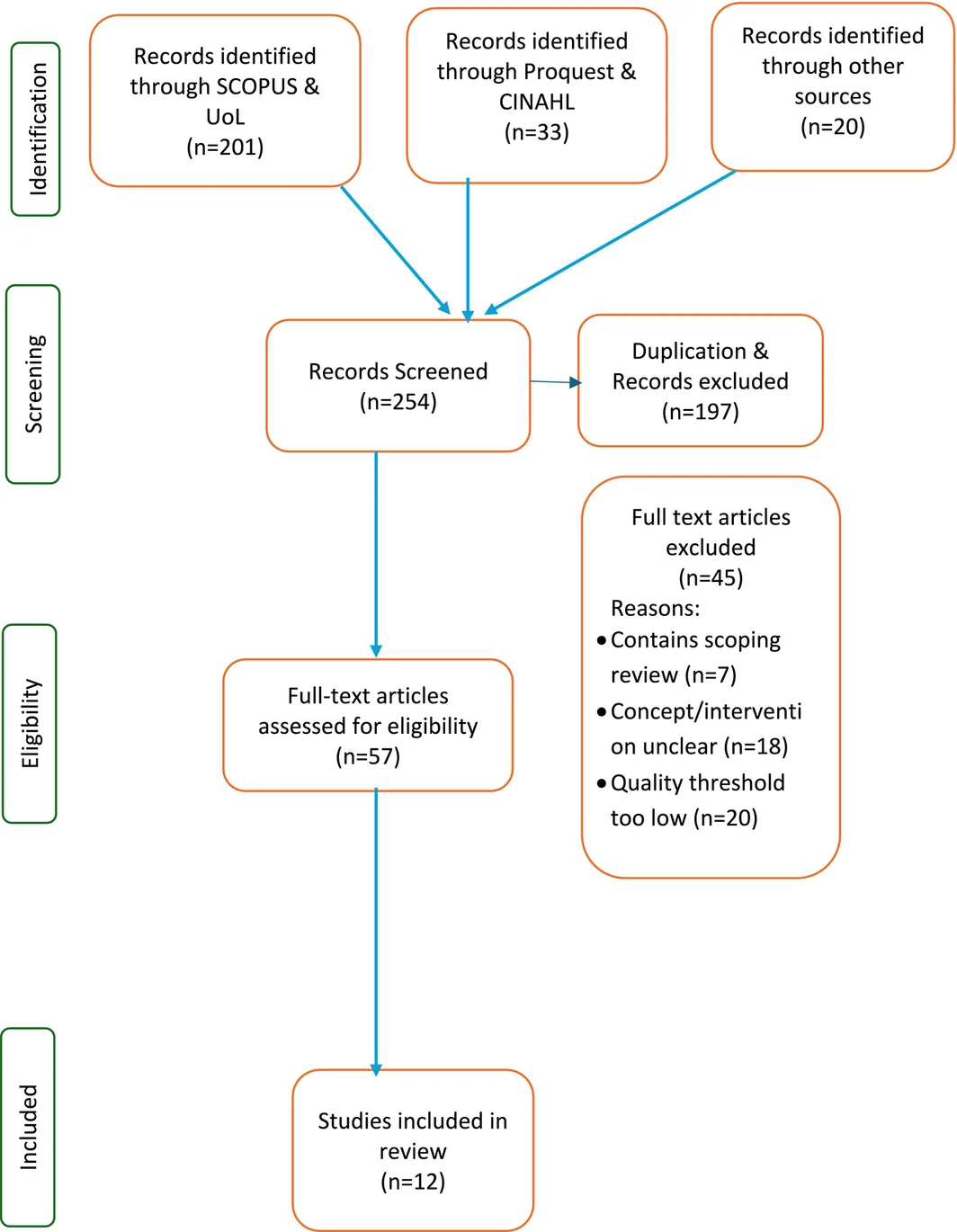
Search outcome flow chart using a PRISMA‐style diagram, adapted from Moher et al. (2009).

Titles and abstracts were screened against the eligibility criteria. Full texts were then reviewed for relevance to the review aims and contribution to understanding de‐escalation, staff training, restrictive‐practice reduction or organisational determinants of aggression and violence. Reference lists of included studies and relevant reviews were searched to identify additional sources. A PRISMA‐style flow diagram was used to summarise the search and selection process (See Figure 1). The flow diagram is included to improve transparency; however, the paper is not presented as a full systematic review. The final empirical evidence base comprised 12 principal studies that directly informed the synthesis. Additional theoretical, policy, implementation and international literature was used to contextualise the analysis, support conceptual interpretation and inform implications for practice and policy.

### 2.3 Appraisal

Given the heterogeneity of the included literature, a single standardised appraisal tool was not considered appropriate. Methodological strengths and limitations were assessed using design‐appropriate appraisal principles informed by established tools, including the Critical Appraisal Skills Programme (CASP) for qualitative studies (Table 1) and the Mixed Methods Appraisal Tool for mixed‐methods research (Appendix B). Appraisal was used to support interpretation of the evidence rather than to determine inclusion or exclusion. Evidence quality was considered alongside conceptual relevance and contribution to understanding relational, capability and contextual determinants of de‐escalation.

**TABLE 1:** Critical appraisal of included studies.

Author(s), year	1	2	3	4	5	6	7	8	9	10	Include?
	Clear aim	Appropriate methodology	Appropriate designs	Appropriate sampling and recruitment	Appropriate data collection	Consideration of research/participant relationship	Consideration of ethical issues	Rigorous analysis	Findings clearly stated	Is the research valuable?	
Berry et al. (2016)	Y	P	Y	Y	Y	Y	Y	Y	Y	Y	Y
Duxbury et al. (2019)	Y	Y	Y	Y	Y	P	Y	Y	Y	P	Y
Goodman et al. (2020)	P	Y	Y	Y	Y	Y	Y	Y	Y	Y	Y
Jalil et al. (2020)	Y	Y	Y	Y	Y	Y	Y	Y	Y	Y	Y
Lavelle et al. (2016)	Y	Y	Y	Y	Y	P	Y	Y	Y	Y	Y
Hallett et al. (2018)	Y	Y	Y	Y	Y	Y	Y	Y	Y	Y	Y
Johnston et al. (2022)	Y	Y	Y	Y	Y	Y	Y	Y	Y	Y	Y
Long et al. (2016)	Y	Y	P	Y	Y	Y	Y	Y	Y	Y	Y
Price et al. (2018)	Y	Y	Y	Y	Y	Y	Y	Y	Y	Y	Y
Renwick et al. (2016)	Y	Y	Y	Y	Y	P	Y	Y	Y	Y	Y
Stewart and Bowers (2014)	Y	Y	Y	Y	Y	P	Y	Y	Y	Y	Y
Wood et al. (2021)	Y	Y	Y	Y	Y	Y	Y	Y	Y	Y	Y

Abbreviations: N/A, not applicable; N, no (criterion not met); P, partially met criterion; Y, yes (fully met criterion).

### 2.4 Data Extraction and Synthesis

Data were extracted on author, year, study design, setting, population, intervention or focus, key findings, limitations and contribution to the synthesis. Extracted material was analysed using a narrative and thematic approach. Initial coding focused on recurrent concepts relating to therapeutic relationships, communication, staff self‐regulation, training, simulation, restrictive‐practice reduction, ward environment, leadership, equity and organisational context. Codes were compared across studies and grouped into higher‐order categories. Through iterative synthesis, three interdependent domains were identified: People, Capability and Context (PCC). These domains were not imposed at the outset but developed through synthesis of the literature. The resulting PCC framework is therefore presented as the conceptual output of the synthesis.

### 2.5 Criteria

1. Clear statement of the research aims.
2. Appropriate study design.
3. Appropriate recruitment or sampling strategy.
4. Appropriate data collection methods.
5. Consideration of researcher–participant relationship or potential bias (where applicable).
6. Ethical considerations adequately addressed.
7. Rigorous data analysis.
8. Clear presentation of findings.
9. Sufficient value and contribution to practice or knowledge.
10. Overall methodological coherence between research question, methods, analysis and interpretation.

## 3 Results

The synthesis identified three interdependent domains shaping the enactment and sustainability of de‐escalation in inpatient mental health care: PCC. These domains are presented below as findings from the synthesis. The People domain captures relational and interactional determinants of de‐escalation; the Capability domain captures workforce knowledge, skills, confidence and learning systems; and the Context domain captures organisational, environmental, equity and system‐level conditions shaping whether de‐escalation can be enacted consistently.

### 3.1 Theme 1: People (Therapeutic Relationships, Communication and Emotional Intelligence)

Nine of the twelve principal empirical studies identified therapeutic relationships, communication, emotional intelligence or other relational processes as key determinants of effective de‐escalation, highlighting the centrality of people. De‐escalation was reported as being shaped by the quality of human interaction. Core elements associated with effective practice included empathy, validation, calm and non‐threatening verbal and non‐verbal communication, appropriate interpersonal distance and continuous risk appraisal (Hallett et al. 2018; Lavelle et al. 2016; Long et al. 2016). Therapeutic relationships characterised by trust, fairness and consistency were described as supporting personalised responses, co‐produced safety planning and the timely use of least restrictive options. In contrast, poorly attuned, inconsistent or emotionally reactive responses were associated with heightened perceptions of threat, erosion of trust and increased likelihood of escalation and coercive intervention (Berry et al. 2016; Goodman et al. 2020; Johnston et al. 2022; Stewart and Bowers 2014).

Emotional intelligence (EI) was identified across the literature as underpinning the enactment of relational practice under conditions of pressure and uncertainty (Johnston et al. 2022). Staff capacity for self‐regulation was associated with calmer engagement and reduced defensive responses, while perspective‐taking and situational awareness supported more accurate interpretation of verbal and behavioural cues. Clearly, non‐threatening communication was linked to reductions in arousal and distress (Goodman et al. 2020; Jalil et al. 2020). These studies described EI as mediating whether relational intent translated into effective de‐escalation in practice. Where escalation progressed despite relational and environmental strategies, or where there was a history of severe violence, the use of pro re nata (PRN) medication was reported as clinically indicated as an adjunctive measure (Hallett 2018). However, several studies cautioned against PRN use that displaced therapeutic engagement. Proportionate and transparent use, with joint review where possible, was associated with preservation of therapeutic alliance and reduced risk of coercive drift (Goodman et al. 2020; Price et al. 2018).

Timing was also identified as a determinant of effectiveness. Incidents were reported to cluster around predictable high‐risk periods, including handovers, mealtimes and medication rounds, during which early, relationally attuned responses were associated with the interruption of escalation trajectories (Duxbury et al. 2019; Johnston et al. 2022; Price et al. 2018; Renwick et al. 2016). Structured post‐incident debriefs were described as supporting reflective practice and team learning, contributing to shared understanding following episodes of heightened risk.

### 3.2 Theme 2: Capability (Standardised Training, Team Simulation and Continuous Learning)

Nine of the twelve principal empirical studies identified workforce capability, skills, confidence and continuous learning as a determinant of effective de‐escalation. De‐escalation training was reported as widely implemented across inpatient mental health services; however, substantial variation was evident in training content, duration and pedagogical approach. While improvements in staff knowledge and self‐reported confidence were frequently described, evidence of sustained reductions in incidents or restrictive practices was inconsistent, particularly where training was delivered in isolation from ward‐based practice and team processes (Duxbury et al. 2019; Jalil et al. 2020; Long et al. 2016).

Studies consistently reported that training programmes incorporating simulation and scenario‐based rehearsal were more closely associated with observable changes in practice than didactic approaches alone. Simulation‐based training enabled teams to rehearse coordinated, real‐time responses within environments that mirrored ward conditions, including role allocation during escalation, leadership of verbal engagement, management of space and bystanders and monitoring of behavioural and physiological indicators (Hallett et al. 2018; Jalil et al. 2020; Long et al. 2016; Price et al. 2018). These approaches were also associated with the development of non‐technical skills, including shared situational awareness, closed‐loop communication, assertive followership and adaptive role switching. Several studies further identified simulation as a mechanism for practising ethical disengagement when verbal strategies were ineffective, supporting trauma‐informed approaches and reducing reliance on physical intervention (Helena Goodman et al. 2020; Hallett 2018).

Evaluation of training effectiveness was most reported using proximal indicators of practice, including the timeliness and quality of de‐escalatory responses, patterns of near‐miss incidents and trends in restraint and seclusion, rather than course completion or certification alone (Johnston et al. 2022). Capability was also shaped by team norms and beliefs. Staff attitudes towards restraint, therapeutic boundaries and shared responsibility for safety were found to influence the application of de‐escalation strategies in practice (Berry et al. 2016; Price et al. 2018). Studies reporting more sustained practice change described training that was supported by protected time, psychologically informed supervision and regular refreshers; in contrast, training gains were reported to diminish in contexts characterised by high workload and staff turnover (Hallett et al. 2018; Johnston et al. 2022; Long et al. 2016).

### 3.3 Theme 3: Context (Staffing, Environment, Leadership and Equity)

Ten of the twelve principal empirical studies identified organisational and environmental context as a key determinant of effective de‐escalation. Rather than functioning as a passive backdrop, organisational context actively shaped the feasibility, consistency and sustainability of therapeutic responses through its influence on staffing, leadership, ward culture, physical environment and opportunities for early intervention. Marked variation in rates of restrictive practice between wards serving comparable populations was frequently attributed to contextual determinants, including staffing levels and skill mix, leadership behaviours, ward design and safety culture (Goodman et al. 2020; Jalil et al. 2020; Lavelle et al. 2016). These factors were reported to influence whether de‐escalation was initiated early, sustained over time or replaced by more reactive and coercive responses (Duxbury et al. 2019).

Adequate numbers of staff with appropriate expertise were associated with earlier engagement, sustained observation and flexible responses to emerging risk (Johnston et al. 2022; Renwick et al. 2016). In contrast, low staffing ratios and high staff turnover were reported to contribute to missed early warning signs, reduced observation quality and compromised situational awareness (Goodman et al. 2020; Price et al. 2018). Skill mix was also highlighted, with wards characterised by higher proportions of experienced mental health nurses, well‐trained support workers and stable teams demonstrating faster recognition of escalation cues, greater confidence in prevention and stronger therapeutic continuity (Berry et al. 2016; Jalil et al. 2020). Several studies noted that, in the absence of these conditions, training interventions were difficult to enact reliably or safely in practice (Hallett and Dickens 2017; Long et al. 2016).

The physical environment was reported as a further contextual influence on agitation and early risk detection. Access to quiet rooms and sensory modulation spaces, clear lines of sight and managed traffic flow were associated with improved emotional regulation and awareness of emerging tensions (Hallett and Dickens 2017; Johnston et al. 2022; Renwick et al. 2016). Conversely, noisy, cluttered or high‐throughput ward environments were linked to heightened staff stress and reduced opportunities for early verbal engagement (Goodman et al. 2020). Predictable routines protected one‐to‐one contact, and access to outdoor space was also described as supporting emotional stability and relational continuity (Berry et al. 2016).

Leadership processes were consistently identified as shaping ward culture and responses to escalation. Leadership behaviours that emphasised psychological safety, early help‐seeking, role clarity and structured communication were associated with greater consistency in de‐escalation practice (Johnston et al. 2022; Price et al. 2018). Post‐incident learning processes focused on reflection and system improvement, rather than blame, were linked to improvements in team coordination and ward climate (Berry et al. 2016; Johnston et al. 2022).

Equity‐related issues were reported across multiple studies. Racialised groups were disproportionately subject to restraint, highlighting the relevance of cultural safety (i.e., care that acknowledges power imbalances and is experienced as safe by those receiving it), access to advocacy and routine monitoring of restrictive practices by protected characteristics (Helena Goodman et al. 2020; Johnston et al. 2022). Wider structural factors, including deprivation, housing insecurity and co‐morbid substance use, were also identified as shaping ward acuity and the feasibility of early engagement (Hallett and Dickens 2017).

### 3.4 The People‐Capability‐Context Framework

The synthesis generated the People‐Capability‐Context framework as a practice‐facing model for understanding how de‐escalation is enacted and sustained in inpatient mental health care. The framework does not propose that PCC are entirely new domains. Rather, its contribution lies in conceptualising de‐escalation as an emergent practice arising from alignment between relational practice, workforce capability and organisational context. Within this framework, People refer to therapeutic interaction, emotional intelligence, communication, relational security (i.e., therapeutic use of interpersonal relationships, professional knowledge and understanding of individuals to anticipate, prevent and respond safely to risk) and service‐user involvement. Capability refers to the knowledge, skills, confidence, simulation, supervision and reflective learning required to translate de‐escalation into practice. Context refers to the staffing, leadership, ward environment, safety culture, equity safeguards and wider service conditions that enable or constrain non‐coercive care.

**FIGURE 2:**
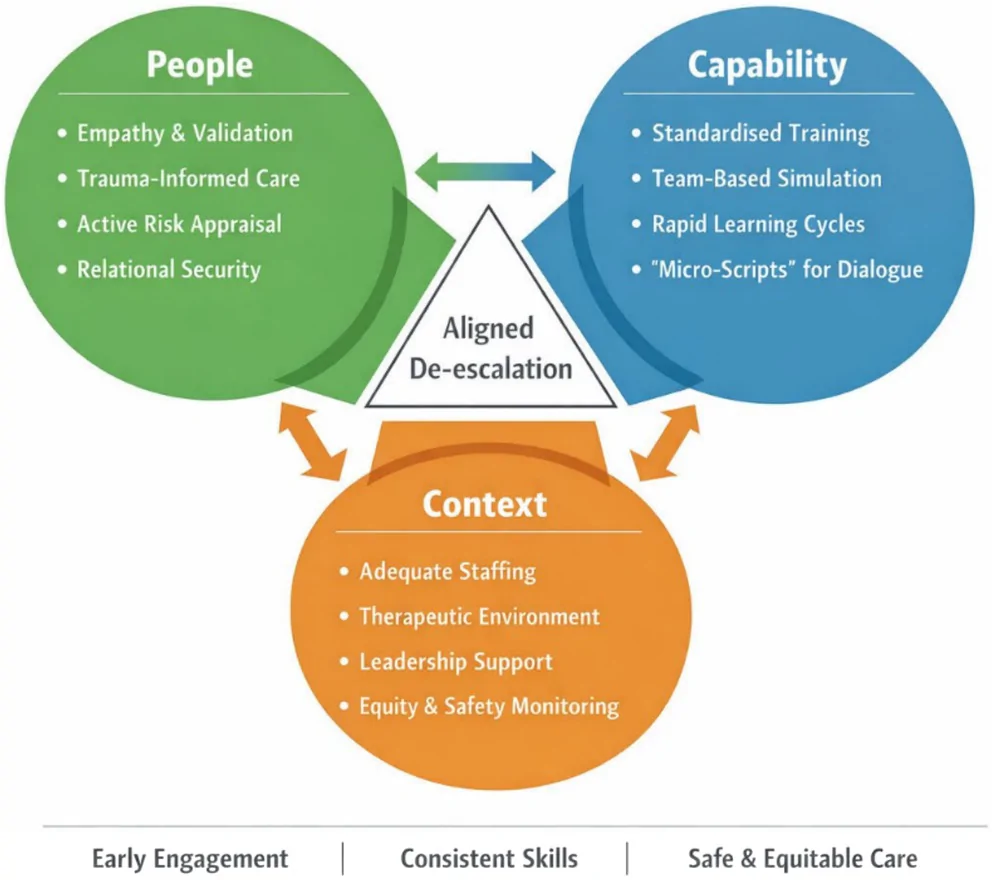
The PCC framework for de‐escalation in inpatient mental health care.

Figure 2 illustrates the People‐Capability‐Context (PCC) framework, showing how alignment across relational practice, workforce capability and organisational context enables reliable de‐escalation within inpatient mental health settings.

## 4 Discussion

This structured critical narrative synthesis generated the PCC framework as a practice‐facing model for understanding de‐escalation and restrictive‐practice reduction in inpatient mental health care. Its novelty does not rest on naming new determinants of practice. Therapeutic relationships, workforce capability, leadership and organisational culture are already recognised as central to patient safety and quality improvement (Braithwaite et al. 2015). The contribution of PCC is to show how these elements need to align in practice. Effective de‐escalation is therefore understood as a product of relational work, workforce capability and organisational conditions operating coherently at the point of care. Restrictive practices, in turn, can be read as indications of misalignment across interacting systems, rather than as failures located only in individual staff behaviour or organisational policy.

### 4.1 Positioning PCC Within Existing Restrictive‐Practice Frameworks

Over the last two decades, several influential frameworks have shaped efforts to reduce restrictive practices. Safewards focuses on reducing conflict and containment through structured ward‐level interventions (Bowers et al. 2015), Six Core Strategies emphasises leadership, organisational change and data‐informed practice (Huckshorn 2005), while trauma‐informed approaches seek to understand distress through adversity and avoid re‐traumatisation (Sweeney et al. 2018). Recovery‐oriented models, the Weddinger Model, MAP and the WHO Quality Rights initiative similarly emphasise collaboration, rights and person‐centred care (Bak et al. 2015; Cole et al. 2020; WHO 2019). The Restraint Reduction Network standards further provide guidance regarding governance, training and least restrictive practice (Restraint Reduction Network 2021). The PCC framework complements rather than replaces these approaches. Existing models primarily specify what organisations should implement. In contrast, PCC seeks to explain why interventions that are effective in one context may fail to translate reliably into another. Its central proposition is that restrictive‐practice reduction is an emergent property arising from alignment between PCC. In this respect, PCC shifts attention from individual interventions towards the coupling of relational, professional and organisational processes required for interventions to function effectively. This aligns with contemporary views of healthcare as a complex adaptive system in which outcomes emerge from interactions between multiple components rather than from isolated interventions (Braithwaite et al. 2018; Greenhalgh and Papoutsi 2018).

### 4.2 Mechanisms of Alignment: How PCC Operates

A central contribution of the framework lies in explaining the mechanisms through which alignment operates. High‐quality therapeutic relationships alone are unlikely to prevent escalation when staffing shortages, overcrowding or weak leadership constrain opportunities for engagement (Baker et al. 2019; Kohanová et al. 2026). Similarly, staff may possess advanced de‐escalation skills but struggle to translate these into practice when psychologically safe learning cultures and supportive organisational conditions are absent (McKeown et al. 2019; Vogt et al. 2025). Conversely, favourable organisational conditions may fail to reduce restrictive practices if teams lack confidence, emotional intelligence or shared approaches to managing distress (Littlejohn 2012; Reilly 2023). PCC therefore conceptualises restrictive practices as manifestations of system misalignment. Workforce shortages, high patient acuity and environmental pressures may reduce opportunities for therapeutic interaction and impair early recognition of distress (Kohanová et al. 2026).

Reduced engagement can increase anxiety among both patients and staff, narrowing available responses and increasing the likelihood of coercive interventions (Krieger et al. 2021; Sweeney et al. 2018). In contrast, when emotionally intelligent communication, psychologically safe teams, effective leadership and supportive environments reinforce one another, early intervention becomes more feasible and escalation trajectories may be interrupted before restrictive measures become necessary (Koutsofta et al. 2025; Reilly 2023). Emerging evidence from real‐time digital monitoring of acute mental health wards supports this systems perspective. WardSonar data demonstrate that safety outcomes arise through dynamic interactions between patient mix, environmental conditions, staffing levels and team communication rather than individual competence alone (Baker et al. 2024). Emerging digital approaches further suggest that alignment across relational, capability and contextual domains may be amenable to prospective monitoring. Situational awareness theories propose that safety depends on the availability of information and the capacity of teams to perceive, interpret and anticipate changing conditions (Munir et al. 2022).

In practice, PCC alignment could be assessed through a small set of complementary indicators across the three domains. People indicators might include service‐user experience of therapeutic engagement, relational safety and involvement in care. Capability indicators could include staff confidence, team psychological safety, de‐escalation competence and reflective learning. Context indicators might include staffing resilience, ward acuity, leadership support, environmental pressures and equity‐sensitive restrictive‐practice rates. Alignment would be indicated when favourable conditions across these domains coincide with effective early intervention and lower reliance on restrictive practices, whereas divergence, for example, high staff competence alongside poor staffing resilience or deteriorating relational safety would signal potential misalignment. On one hand, this conceptualisation is consistent with implementation science approaches such as consolidated framework for implementation research (CFIR) (Damschroder et al. 2022), which emphasises the need for meaningful interactions between individuals, implementation processes and organisational context rather than isolated determinants of practice. On the other hand, normalisation process theory (NPT) (May and Finch 2009) could examine how PCC‐informed practices are understood, enacted and embedded in routine clinical work. Bearing in mind this need for alignment, early changes in ward activity, environmental pressures, communication patterns or patient acuity may provide signals of emerging risk before escalation becomes overt. Such approaches raise the possibility that de‐escalation may be strengthened through systems capable of supporting shared situational awareness and anticipatory responses rather than relying solely on retrospective incident reporting. Such findings support a move away from individualised explanations of de‐escalation failure towards understanding safety as an emergent property of interconnected systems.

### 4.3 PCC as a Systems‐Oriented Model of Safety

The framework aligns with contemporary theories of healthcare as a complex adaptive system, where outcomes emerge through interactions between people and environments rather than linear cause‐and‐effect relationships (Braithwaite et al. 2018; Greenhalgh and Papoutsi 2018). Traditional approaches to patient safety have focused on identifying errors and preventing adverse events (Hoffmann and Rohe 2010; Jayaram 2014). More recent Safety‐II perspectives emphasise understanding how successful outcomes are routinely achieved despite changing conditions and operational pressures (Hollnagel 2017; Vanderhaegen 2015). From this perspective, successful de‐escalation represents a form of everyday clinical resilience. Leadership, ward culture, staffing and physical environments should therefore be viewed as active determinants of safety rather than contextual background factors (Ridelberg et al. 2014; Zamani et al. 2023). This interpretation moves beyond viewing aggression and coercion solely as behavioural phenomena and instead positions them within broader socio‐technical systems (Vincent and Amalberti 2016).

Recent international evidence further demonstrates substantial discrepancies between how healthcare professionals conceptualise restrictive practices and how they are ultimately documented within organisations (Belayneh et al. 2026). Reporting behaviours are influenced by workload, staffing levels, institutional priorities and local reporting cultures (Belayneh et al. 2026; Kohanová et al. 2026). This reinforces the PCC proposition that context functions as an active determinant of practice rather than a passive backdrop and that organisational conditions shape the reliability of restrictive‐practice reduction efforts.

### 4.4 Relational Care, Lived Experience and Co‐Produced Safety

The prominence of relationships within the framework reflects longstanding traditions within mental health nursing (Beyene et al. 2026; Bohmer 2024). Peplau's interpersonal theory and contemporary person‐centred approaches position healing as fundamentally relational rather than procedural (Adler 2020; McCormack and McCance 2016). Mental health nursing scholarship similarly emphasises social connectedness and therapeutic engagement as mechanisms through which recovery and safety are achieved (McKeown et al. 2020). Service users appear particularly sensitive to issues relating to autonomy, dignity and psychological safety, whereas clinicians' interpretations are more strongly influenced by legal frameworks and procedural requirements (Akther et al. 2019). These differences suggest that the meaning and impact of restrictive interventions cannot be understood solely through objective events but through how they are experienced and interpreted. Effective de‐escalation depends on shared meaning‐making, culturally safe communication and collaborative responses to distress rather than unilateral attempts to manage behaviour (Sweeney et al. 2018). Such interpretations are consistent with recovery‐oriented and person‐centred approaches that position service users as active participants rather than passive recipients of risk management (Daguman et al. 2026; Sweeney et al. 2018).

### 4.5 Capability Beyond Training: Towards Learning Systems

The findings of this review also challenge assumptions that de‐escalation capability can be achieved through training alone. Educational interventions frequently improve knowledge and confidence but do not necessarily produce sustained behavioural change (McCluskey et al. 2005). Contemporary learning theories emphasise reflection, feedback and experiential learning as mechanisms through which knowledge is translated into practice (Kolb 2013). Similarly, team training and simulation‐based approaches improve collective performance when embedded within psychologically safe learning cultures (Baby et al. 2018). Emerging work suggests that practice‐proximal evaluation, simulation‐rich rehearsal and continuous learning embedded within ward routines may strengthen the transfer of knowledge into practice (Simmons et al. 2023; Urheim et al. 2013). Capability should therefore be understood as a collective property of teams rather than an attribute possessed by individuals.

### 4.6 Equity and Structural Determinants

One of the strengths of PCC lies in positioning equity as integral to patient safety rather than as an additional consideration. Evidence from the United Kingdom consistently demonstrates disproportionate rates of detention, restraint and coercive intervention among Black and racialised communities (Frankova 2020; Joyes and Jordan 2022; Rabab et al. 2020). Structural racism, institutional practices and unequal access to resources contribute to these disparities (Bailey et al. 2017). Cultural safety provides an important lens because it recognises power imbalances and positions safety as defined by those receiving care rather than solely by those providing it (Curtis et al. 2019). UK policy increasingly reinforces this equity imperative. The Independent Review of the Mental Health Act identified racial disproportionality in detention and emphasised autonomy, least restriction and therapeutic benefit (Wessely et al. 2018). The Patient and Carer Race Equality Framework (PCREF) translates these principles into organisational expectations for co‐production, accountability and ethnicity‐informed monitoring of inequalities, including use of force (NHS England 2023). Complementing this, CQC findings on restraint, seclusion and long‐term segregation highlight the importance of person‐centred care, workforce capability and system‐level accountability (Care Quality Commission 2020). These policy developments reinforce PCC by locating equity across relationships (People), culturally responsive practice (Capability) and organisational governance (Context).

PCC proposes that reducing racialised coercion requires alignment across all three domains. Culturally safe relationships, anti‐racist workforce capability and organisational accountability are mutually reinforcing. Failure within any one domain risks perpetuating inequities even when overall restrictive‐practice rates decline. Poverty, housing insecurity, social exclusion and deprivation shape pathways into inpatient care and influence the complexity of needs encountered by clinical teams (Jaffee et al. 2025; WHO 2019). Restrictive‐practice reduction should therefore be understood not solely as a ward‐level challenge but as one requiring coordination across healthcare, social care, housing and commissioning systems.

### 4.7 Relevance for Clinical Practice

Table 2 translates PCC into practical actions and indicators. These are not intended as standalone interventions; their value lies in supporting coordinated improvement across all three domains. The argument advanced in this paper translates into a focused set of high‐leverage actions that services can implement immediately, while building towards a more coherent national framework for de‐escalation. A central priority is to make emotional intelligence and trauma‐informed communication explicit and assessable competencies within Prevention and Management of Violence and Aggression (PMVA) training, supervision and appraisal. This can be reinforced through brief, structured debriefs following escalation events or near misses, supporting reflective learning, consolidating skills and reducing drift from relational practice.

**TABLE 2:** Applying the People‐Capability‐Context (PCC) framework in clinical practice.

PCC domain	Priority actions	Indicators of progress
People	Embed emotional intelligence, trauma‐informed communication, collaborative safety planning and culturally safe engagement within everyday practice. Involve service users in care planning, post‐incident reviews and restrictive‐practice reduction initiatives	Improved patient‐reported trust and therapeutic relationships; increased use of collaborative safety plans; reduced complaints relating to communication, dignity or coercion
Capability	Standardise core de‐escalation competencies nationally while allowing local adaptation. Implement simulation‐based learning, structured debriefing, reflective practice and regular refresher training	Improved staff confidence and competence; increased participation in simulation training; improved quality and timeliness of de‐escalation responses; reduced reliance on restrictive interventions
Context	Ensure adequate staffing, stable skill mix, visible leadership, psychologically safe team cultures and access to therapeutic and sensory spaces. Monitor restrictive‐practice data through an equity lens and strengthen organisational accountability	Reduced rates of restraint, seclusion and PRN use; improved staff retention; enhanced psychological safety; reduced disparities in restrictive practice use across protected characteristics

Standardising the core components of de‐escalation training at a national level, while retaining flexibility for local adaptation, represents a further priority. Training should be complemented by regular team‐based simulation and scenario‐based huddles that rehearse coordinated responses under realistic conditions. Such rehearsal clarifies role allocation during escalation, including leadership of verbal engagement, management of space and bystanders and safe transfer of responsibility when verbal strategies are insufficient. Evaluation of training impact should prioritise practice‐proximal indicators such as the timeliness and quality of de‐escalatory responses, patterns of incident clustering and trends in restraint and seclusion rather than relying solely on course completion.

Equally important is the intentional design of inpatient environments to support calm interaction and early engagement. Clear sightlines, access to quiet or sensory modulation spaces, managed traffic flow and protected one‐to‐one contact all facilitate prevention. Adequate staffing and visible, relational leadership are essential to prioritise early intervention over control and align risk management with therapeutic care. Applying an equity lens by default is integral to safety, requiring routine disaggregation of restrictive practice data, access to cultural safety training and advocacy and meaningful service‐user involvement in care planning. Together, these actions operationalise a practice‐grounded approach to de‐escalation and outline the foundations of a coordinated, system‐wide response.

## 5 Conclusions

De‐escalation can displace coercive practices at scale only when people, capability and context are purposefully aligned. At its core lies emotionally intelligent, trauma‐informed nursing practice that builds trust, accurately reads cues and communicates with clarity under pressure. For such practice to be sustained, organisations must move beyond episodic training and invest in consistent, immersive learning approaches that transform incidents and near misses into opportunities for reflection and growth. Simulation‐rich, team‐based rehearsal strengthens collective performance, embedding prevention as a routine and shared responsibility rather than a reactive response to crisis. Equally, effective de‐escalation depends on environments intentionally designed to support calm interaction and early engagement, alongside leadership that models proactive behaviours, fosters psychological safety and places equity at the centre of safety and quality agendas. The PCC model offers a pragmatic framework for understanding and aligning these interdependent elements in everyday inpatient care.

Adoption of a coordinated national approach, one that specifies core de‐escalation competencies, supports ward‐level enablers and incorporates equity‐aware metrics, has the potential to reduce unwarranted variation, strengthen safety systems and improve experiences for both patients and staff. Aligning de‐escalation practice in this way is essential to making rights‐respecting, non‐restrictive care more reliable and sustainable across UK inpatient mental health services. While the PCC model is developed in the UK context, its principles are likely transferable to comparable international inpatient systems, subject to adaptation to local legal, workforce and funding contexts.

## 6 Limitations

The PCC framework offers a heuristic for improving inpatient mental health practice but has notable limitations. Developed through a structured critical narrative synthesis of empirical, theoretical, policy and practice literature, it is grounded in curated evidence rather than primary research and has yet to be prospectively tested. Its explanatory, predictive and implementation value remains to be established. Evidence integration involved author judgement, using heterogeneous sources of varied qualitative studies, service evaluations, observational research, policy and reviews, despite transparent search and appraisal procedures. The three‐domain model simplifies the dynamic, reciprocal relationships between people, capability and context, and may omit key influencing factors. While equity and structural determinants are incorporated, the framework was not co‐produced with those with lived experience of restrictive practices. Future refinement should prioritise lived‐experience input to define safe, culturally responsive, non‐coercive care. PCC reflects UK inpatient mental health services and policy, and international application will require adaptation to local laws, workforce structures, funding and cultural contexts. Future research should focus on operational measures, testing its implementation utility and evaluating whether alignment across its domains improves patient, staff and service outcomes.

## Author Contributions

Ndukwe Walter Ugwuocha led the conceptual development and undertook the primary writing of the essay. Chen Sheoko, as the Restrictive Practice Reduction Consultant, contributed valuable practice insights, contextual expertise and institutional support. Alexander Challinor and Deborah Aluh contributed to reviewing, refining and enriching the final manuscript. Oladayo Bifarin and Simon Neilson provided academic supervision, critical review and guidance throughout the development of the manuscript. All authors listed meet the authorship criteria according to the latest guidelines of the International Committee of Medical Journal Editors. All authors are in agreement with the manuscript.

## Funding

The authors have nothing to report.

## Conflicts of Interest

Oladayo Bifarin is a National Institute for Health and Care Research Leader; however, the views expressed in this article are those of the author and not necessarily those of NIHR or the Department of Health and Social Care.

## Data Availability

The data that support the findings of this study are available on request from the corresponding author. The data are not publicly available due to privacy or ethical restrictions.

## Appendix A Search String

The core Boolean search string was adapted for each database as follows: (‘de‐escalation’ OR ‘verbal de‐escalation’ OR ‘de‐escalation techniques’ OR ‘conflict de‐escalation’ OR ‘aggression management’ OR ‘violence prevention’ OR ‘restraint reduction’ OR ‘restrictive practice reduction’)

AND

(‘staff training’ OR education OR ‘training programme’ OR ‘training program’ OR simulation OR ‘skills training’ OR ‘staff development’ OR ‘workforce capability’)

AND

(‘inpatient mental health’ OR ‘psychiatric inpatient’ OR ‘acute mental health’ OR ‘forensic mental health’ OR ‘secure mental health’ OR ‘mental health ward’ OR ‘psychiatric ward’)

AND

(aggression OR violence OR conflict OR agitation OR restraint OR seclusion OR containment)

A supplementary search was also used to identify literature on organisational and contextual determinants:

(‘inpatient mental health’ OR ‘psychiatric inpatient’ OR ‘mental health ward’ OR ‘forensic mental health’ OR ‘secure mental health’)

AND

(aggression OR violence OR conflict OR agitation OR restraint OR seclusion OR ‘restrictive practice’)

AND

(de‐escalation OR ‘violence prevention’ OR ‘restraint reduction’ OR containment)

AND

(staffing OR leadership OR ‘ward environment’ OR ‘ward culture’ OR ‘organisational culture’ OR ‘safety culture’ OR training OR education)

## Appendix B Methodological Appraisal of the Principal Empirical Studies Informing the People‐Capability‐Context (PCC) Framework

**Table:**

Study	Study design	Appraisal approach	Overall methodological judgement	Principal strengths	Principal limitations	Contribution to PCC synthesis
Berry et al. (2016)	Qualitative interview study	CASP Qualitative Checklist	High	Rich service‐user and staff perspectives; rigorous thematic analysis; strong credibility	Single NHS setting; transferability may be limited	People; context
Stewart and Bowers (2014)	Qualitative study	CASP Qualitative Checklist	Moderate‐high	Detailed exploration of therapeutic relationships and communication	Small sample; limited diversity of participants	People
Lavelle et al. (2016)	Systematic review of qualitative evidence	CASP Systematic Review principles	High	Comprehensive synthesis of de‐escalation experiences; transparent review methods	Heterogeneity between included studies	People
Long et al. (2016)	Systematic review	CASP Systematic Review principles	High	Comprehensive review of de‐escalation training interventions; transparent methodology	Considerable methodological heterogeneity prevented strong conclusions	Capability
Renwick et al. (2016)	Observational/service evaluation	CASP Cohort principles	Moderate	Real‐world ward observations; clinically relevant findings	Single service; limited control for confounding	People; context
Hallett and Dickens (2017) and Hallett et al. (2018)	Mixed‐methods evaluation	MMAT (2018)	High	Integration of qualitative and quantitative findings; strong practice relevance	Conducted within one organisation; limited longitudinal evaluation	People; capability
Price et al. (2018)	Mixed‐methods implementation evaluation	MMAT (2018)	High	Strong focus on implementation and reflective practice; triangulation of data	Limited generalisability beyond participating services	Capability; context
Duxbury et al. (2019)	Multi‐site implementation study	MMAT (2018)	High	Large‐scale implementation; organisational perspective; robust mixed‐methods design	Variable fidelity between sites	Capability; Context
Goodman et al. (2020)	Mixed‐methods study	MMAT (2018)	High	Examined organisational culture, staffing and restrictive practice using multiple data sources	Cross‐sectional design limits causal inference	People; context
Jalil et al. (2020)	Mixed methods/service evaluation	MMAT (2018)	Moderate‐high	Practical evaluation of training and simulation; good integration of findings	Small sample; short follow‐up period	Capability; context
Wood et al. (2021)	Qualitative implementation study	CASP Qualitative Checklist	High	Detailed exploration of implementation barriers and facilitators	Single organisational context	Capability
Johnston et al. (2022)	Integrative review	CASP Review principles	High	Broad synthesis of contemporary de‐escalation evidence; strong conceptual analysis	Included studies varied considerably in design and quality	People; capability; context

*Note:* Methodological appraisal was undertaken using design‐appropriate critical appraisal principles. Qualitative studies were appraised using the Critical Appraisal Skills Programme (CASP) Qualitative Checklist. Mixed‐methods studies were appraised using the Mixed Methods Appraisal Tool (MMAT) Version 2018 (Hong et al. 2018). Systematic and integrative reviews were appraised using CASP review principles. Appraisal informed interpretation of findings rather than study inclusion or exclusion. Overall methodological judgements represent the authors' synthesis of study quality, methodological rigour and relevance to the review question.
